# Van Houten's Cocoa v. Whole Meal Bread

**Published:** 1887-11-19

**Authors:** F. O. Morris

**Affiliations:** Nunburnholme Rectory, Hayton, York


### THE EDITOR'S LETTER-BOX.

### VAN HOUTEN'S COCOA v. WHOLE-MEAL BREAD.

In Saturday se'ennight's numbei of The Hospital you highly commend Van Houten's Cocoa, and speak of it as better than any other. With this opinion I quite agree; but you gave as the reason of it—if I remember right, for I gave the copy away to a M.D.—that only the inside of the berry was ground up, and not the outside husk with it. With this also I should always, all along, have naturally agreed, meaning only the nut, and not the shell of it. But our good friends, the Vegetarians, recommend to us, in the strongest possible manner, on the exactly opposite ground, the "whole-meal" bread, as being made of the whole of the grain of wheat, husk and all. "Who shall decide when doctors disagree?" I shall be glad of your decision on the point, as I doubt not many other persons would be too, as the subject is one of daily importance to every one. F. O. Morris.

Nunburnholme Rectory, Hayton, York.

[Whole meal bread may be more pleasant for some persons than bread made of flour from decorticated wheat, but it is neither so rich nor so highly nutritive—vegetarians notwithstanding. It is a general rule, well known even to the most elementary botanists, that the seeds of plants consist of a much more highly azotised substance than the husks or shells. The more highly nitrogenous the plant, the more highly nutritious is it, and the part of the plant which contains the largest proportion of nitrogenous matter is always the germinating portion that is, the actual seed. Hence the superior richness of cocoas and chocolates prepared from the seeds alone.—Ed. T. H.]

